# Psychometric Evaluation of the TWente Engagement with Ehealth Technologies Scale (TWEETS): Evaluation Study

**DOI:** 10.2196/17757

**Published:** 2020-10-09

**Authors:** Saskia Marion Kelders, Hanneke Kip, Japie Greeff

**Affiliations:** 1 Center for eHealth and Wellbeing Research Department of Psychology, Health, and Technology University of Twente Enschede Netherlands; 2 Optentia Research Focus Area North-West University Vanderbijlpark South Africa; 3 School of Computer Science and Information Systems Faculty of Natural and Agricultural Sciences North-West University Vanderbijlpark South Africa

**Keywords:** engagement, attrition, eHealth, adoption, adherence, questionnaire, scale validation, digital health interventions

## Abstract

**Background:**

Engagement emerges as a predictor for the effectiveness of digital health interventions. However, a shared understanding of engagement is missing. Therefore, a new scale has been developed that proposes a clear definition and creates a tool to measure it. The TWente Engagement with Ehealth Technologies Scale (TWEETS) is based on a systematic review and interviews with engaged health app users. It defines engagement as a combination of behavior, cognition, and affect.

**Objective:**

This paper aims to evaluate the psychometric properties of the TWEETS. In addition, a comparison is made with the experiential part of the Digital Behavior Change Intervention Engagement Scale (DBCI-ES-Ex), a scale that showed some issues in previous psychometric analyses.

**Methods:**

In this study, 288 participants were asked to use any step counter app on their smartphones for 2 weeks. They completed online questionnaires at 4 time points: T0=baseline, T1=after 1 day, T2=1 week, and T3=2 weeks. At T0, demographics and personality (conscientiousness and intellect/imagination) were assessed; at T1-T3, engagement, involvement, enjoyment, subjective usage, and perceived behavior change were included as measures that are theoretically related to our definition of engagement. Analyses focused on internal consistency, reliability, and the convergent, divergent, and predictive validity of both engagement scales. Convergent validity was assessed by correlating the engagement scales with involvement, enjoyment, and subjective usage; divergent validity was assessed by correlating the engagement scales with personality; and predictive validity was assessed by regression analyses using engagement to predict perceived behavior change at later time points.

**Results:**

The Cronbach alpha values of the TWEETS were .86, .86, and .87 on T1, T2, and T3, respectively. Exploratory factor analyses indicated that a 1-factor structure best fits the data. The TWEETS is moderately to strongly correlated with involvement and enjoyment (theoretically related to cognitive and affective engagement, respectively; *P*<.001). Correlations between the TWEETS and frequency of use were nonsignificant or small, and differences between adherers and nonadherers on the TWEETS were significant (*P*<.001). Correlations between personality and the TWEETS were nonsignificant. The TWEETS at T1 was predictive of perceived behavior change at T3, with an explained variance of 16%. The psychometric properties of the TWEETS and the DBCI-ES-Ex seemed comparable in some aspects (eg, internal consistency), and in other aspects, the TWEETS seemed somewhat superior (divergent and predictive validity).

**Conclusions:**

The TWEETS performs quite well as an engagement measure with high internal consistency, reasonable test-retest reliability and convergent validity, good divergent validity, and reasonable predictive validity. As the psychometric quality of a scale is a reflection of how closely a scale matches the conceptualization of a concept, this paper is also an attempt to conceptualize and define engagement as a unique concept, providing a first step toward an acceptable standard of defining and measuring engagement.

## Introduction

In eHealth, the use of technology to support health and wellbeing (a well-documented issue) is nonadherence. Especially within digital health interventions (DHIs), users often do not use the offered technology the way that the developers of the technology intended, which is referred to as nonadherence [1,2]. Examples are participants not completing all lessons within a mental health intervention, not using a step counter app on a daily basis, or not using all functions within a diabetes management system. Developers and researchers often assume that there is a dose-response relationship: for people who use a technology more and who adhere to the technology, the positive effects are greater. There is some evidence to support this assumption [3], but many DHIs do not show this relationship [4]. It has been argued that this has to do with the way adherence is conceptualized and measured (eg, with just the number of logins) [4,5]. However, more importantly, it has also been posited that the reasons why people use a technology might be more important in predicting effectiveness than the frequency or duration of use. Research shows that when people use DHIs because they feel involved or are able to identify themselves with the intervention, these DHIs are more likely to be effective for these people [6,7].

When looking at the reasons behind DHI use, the concept of engagement often emerges as a predictor for effectiveness [8-10]. In a broad sense, engagement is often seen as how involved or occupied someone is with something, and as something that is related to a positive outcome, such as effectiveness. For example, in health care, patient engagement has been shown to be related to better health outcomes [11], and in organizations, work engagement is related to better performance [12]. In relation to DHIs, engagement has also been posited as related to better outcomes in terms of more effective interventions [9,13,14].

It is important to note the conceptual difference between adherence and engagement. Adherence only says something about the objective usage of a DHI and indicates whether or not a participant uses the DHI as was intended by the developers [5]. In some, but not all, conceptualizations of engagement, the usage of a DHI is part of engagement [8]. Moreover, in all conceptualizations of engagement, it encompasses other aspects as well, which refer more to the reasons behind using a DHI. This implies that a participant might be engaged but not adherent. For example, someone believes that using an app is very helpful to reach one’s goals (highly engaged) but feels that using the app once a week rather than the intended usage of once a day already helps to reach those goals (nonadherent). A participant may also be adherent but not engaged when, for example, using an app as intended because a researcher or therapist has asked them to, rather than due to a feeling that the app is personally beneficial. While the conceptual differences between engagement and adherence might be understood, within the context of DHIs, a shared understanding and definition of engagement is missing.

In order to gain more insight into how engagement can be defined and conceptualized within the context of DHIs, a recent review looked at the definitions and components of engagement in various domains such as students, health, and digital engagement [8]. This review concluded that engagement contains emotional, behavioral, and cognitive components. Until now, this is not fully reflected in the definitions for DHI engagement, where in many cases, engagement is defined as only a behavioral component (for example, only as the usage of a DHI). This problem of a very narrow definition of engagement that does not encompass the full breadth of the concept is acknowledged by others. For example, a review on engagement in DHIs concluded that engagement should be characterized as the extent of usage and a subjective experience characterized by attention, interest, and affect [9]. However, that review also found that the majority of articles included only viewed engagement in behavioral terms, that is, as usage. Yet the definition chosen in that review also does not seem to fully encompass the emotional, behavioral, and cognitive components of engagement. The field of engagement to DHIs still needs a “clear, tailored, and domain-specific definition of the construct, which captures the associated emotional, behavioral, and cognitive components present within the given context” [8].

In order to gain insight into the content of these components, we interviewed self-proclaimed engaged users of health apps to study what they view as being engaged [13,15]. Behavioral engagement seemed to focus more on having a routine in using the technology and making it a part of daily life than about the frequency of using it. In this case, the quality of the behavior (being a routine and costing little effort) was more important than the frequency of the behavior (usage), which also shows the difference between adherence and engagement. Cognitive engagement with a DHI was found to be prominently related to the goals of the users. People have to think that the technology is useful for them and that it increases their ability to achieve their own goals. This is related to the importance of personal relevance for engagement, as stated in earlier studies on DHIs [6,7,16], and is also similar to the “attention and interest” part of the definition of engagement of Perski et al [9]. Lastly, the study found that affective engagement played a role for every participant but was less salient in most cases. Interestingly, affect was not only focused on feelings towards the technology itself but also on achieving goals, differing from the concept of user engagement [17]. Furthermore, although most participants mentioned positive affect when achieving goals, negative affect also seemed to play a role. Participants experienced frustration when not achieving their goals, and for some, this may enhance their motivation to go on. Finally, identity seemed to play a role in affective engagement: users need to be able to identify in some way with the technology and what it stands for.

A recent systematic review has identified various ways to assess (a form of) engagement to DHIs, such as using qualitative methods, self-report scales, ecological momentary assessments, and system usage data [14]. However, most of these methods assess only a specific form or component of engagement. The study posited that self-report scales might be the most accessible way to gain a more nuanced view of engagement in larger samples, especially when attempting to include the subjective experience. However, most existing self-report scales are created for measuring user engagement with technologies such as e-commerce websites or video games [14]. In these contexts, the goals of users are different than in DHIs. Engagement with DHIs seems to be needed at 2 levels: engagement with the technology itself, and engagement with the health behavior the technology aims to improve [10]. This is in contrast to, for example, user engagement with a shopping website, which involves only engagement with the technology and not with another offline behavior. This makes these existing scales less applicable to eHealth technologies such as DHIs. Another issue with many existing user-engagement scales is that they often include attributes that predict engagement but are not, in itself, part of engagement; this applies, for example, to aesthetic appeal or usability, which raises validity concerns [14].

Within the aforementioned systematic review paper on measuring engagement, 2 scales specifically targeted at engagement with eHealth technologies such as DHIs were identified [14]. The first scale, the eHealth engagement scale, showed adequate internal reliability and predictive validity on, amongst other things, retention of information and intentions to change [18]. However, conceptually, this scale seems to be more focused on engagement with health-related information than acting on that information, and thus on behavior change, as is our focus [9,13,14]. Moreover, this scale is an example of a scale that includes attributes that might predict engagement but are not part of the engagement itself. For example, the scale includes an assessment of the credibility of the technology, which may be a predictor of engagement but is not something that is seen as part of engagement itself. The second scale, the Digital Behavior Change Intervention Engagement Scale (DBCI-ES), was developed based on a broader definition of engagement, including both behavior and a subjective experience that might encompass cognitive and affective components of engagement. However, 2 validation studies showed that the psychometric properties of this scale are somewhat problematic [19,20]. The main issues seem to lie in the combination of the subjective and objective engagement measure, and in the discriminant and criterion validity. The experiential part of the scale (DBCI-ES-Ex) did show predictive validity, but this was not assessed on outcomes (such as changed drinking behavior) but on subsequent login. If engagement is seen as important for effectiveness, even more so than usage and adherence, then it should predict outcomes and not usage.

Based on our previous work on defining the concept of engagement, we developed another scale: the TWente Engagement with Ehealth and Technologies Scale (TWEETS) [15]. This scale was developed based on the interview study with engaged health app users that was discussed earlier [13], and on the systematic review of the concept of engagement in different domains [8]. The scale employs a definition of engagement that incorporates behavior, cognition, and affect, as is common in other fields of research where engagement is used [8,21]. In the TWEETS, engaged behavior includes the existence of a routine in which individuals use the technology, low effort required to use the technology, and technology usage that is not fixed but may fluctuate to fit with the needs of the current moment. Cognitive engagement is related to the technology being able to support and motivate people in reaching their goals, such as the goal of improving one’s wellbeing. Moreover, it entails that engaged users are willing to spend mental effort in using a DHI because it helps them achieve their goals, and they are intrinsically motivated. Affective engagement is related to emotions that people feel when seeing their progress in the DHI, or a lack thereof, and related to emotions such as enjoyment felt when using the technology itself. Lastly, it entails identity: engaged users seem to identify themselves in some way with the technology or with the goal of the technology.

In order for this new scale to be of use, its internal consistency and reliability should be examined. Moreover, assessment is needed for whether the scale measures a unique concept and is sufficiently different from other concepts such as involvement and adherence. As engagement is a relatively new concept without an existing gold-standard assessment, there is no criterion to relate it to, contrary to how the validation of the DBCI-ES was set up [19,20]. In that study, usage was seen as a criterion; however, since engagement is more than just usage [9,10,14,15], we feel that this does not do justice to the full concept of engagement. At the same time, there are concepts that, while different, are related and should correlate with a measure of engagement, and there are concepts that should reflect something different than engagement, and therefore should not be correlated. Specifically, engagement is conceptually related to concepts like involvement (how meaningful a product is for an individual) and enjoyment (the action or state of deriving gratification from an object) [7,22,23]. Therefore, it is expected that engagement correlates with these aspects, but that this correlation is moderate.

Another question that bears discussion is whether engagement is, and should be, related to the usage of a system. Based on the conceptualization of engagement as employed in the TWEETS, it is expected that engagement is not strongly related to the number of times individuals use a DHI. This is similar to what was found in the psychometric evaluation of the DBCI-ES [20]. Furthermore, engagement should reflect a more goal-oriented or intrinsically motivating reason for using a DHI, instead of, for example, using something because you are inclined to do so due to your personality [9,13]. Therefore, engagement should not be correlated to personality traits as conscientiousness and intellect/imagination. Lastly, it is important to assess whether the measure of engagement predicts future outcomes such as behavior change and clinical measures, as it is theorized that engagement influences outcomes and not the mere usage of a system.

The current study was carried out in the context of students using step counter apps. The main goal is to evaluate the psychometric properties of the TWEETS (internal consistency and reliability; and convergent, divergent, and predictive validity). As a secondary exploratory objective, the psychometric properties of the TWEETS were compared to those of the experiential part of the DBCI-ES (DBCI-ES-Ex). We chose not to include the behavioral subscale due to the finding that this behavioral subscale may not be a valid indicator of (behavioral) engagement [20].

## Methods

### Design

For this study, participants were asked to use any step counter app on their smartphones for 2 weeks. Because the study aims at exploring the psychometric properties of the TWEETS and not, for example, how engaging a specific DHI is, the focus on students and a step counter app was deemed feasible and appropriate. Furthermore, we aimed at studying the TWEETS in an ecologically valid way and, therefore, opted to use existing step counter apps instead of, for example, a dedicated research app. Participants were asked to fill out an online questionnaire at 4 time points: T0=baseline, T1=after 1 day, T2=1 week, and T3=2 weeks. This study was approved by the ethical committee of the University of Twente (application number 18881).

At T0, demographics (gender, age, nationality, and employment status) and personality were assessed; at the other 3 time points, aspects related to the use of the step counter app were measured (engagement, involvement, enjoyment, usage, and perceived behavior change). This allowed us to perform the analyses needed to assess the internal consistency and reliability, and the convergent, divergent, and predictive validity of the engagement scales. Convergent validity refers to the degree to which measures of constructs that theoretically *should* be related, *are* related. To assess convergent validity, we chose to correlate the engagement scales to measures that are deemed to be related to behavioral, cognitive, and affective engagement. For behavioral engagement, we decided to use frequency of use and adherence as theoretically related concepts, as these are often posited as part of, or related to, engagement [9,14]. For cognitive engagement, involvement was chosen as the theoretically related concept, as involvement captures the personal relevance of an object (in this case, a DHI), which is often seen as an important part of engagement [6,7,9,16]. For affective engagement, enjoyment was chosen as the theoretically related concept because affective engagement is often seen as a reflection on how much users enjoy using the DHI [22,24].

Divergent validity refers to whether concepts that should theoretically not be related are not related. As engagement should reflect the position of an individual toward a DHI (and not a general trait or an individual), we chose to use personality traits as the concepts for divergent validity. Specifically, the personality traits of conscientiousness and intellect/imagination were used because they might reflect a somewhat similar disposition as engagement but are still theoretically unrelated. Conscientiousness, or being diligent, might lead participants to take the task of using the DHI very seriously and to, therefore, possibly use a DHI more [25]. However, this is a different reason for using a DHI and should thus not be related to engagement. Intellect/imagination—a person's preference for imaginative, artistic, and intellectual activities [26]—could also lead to participants using a DHI more, as many DHIs also involve some degree of intellectual activity. However, as a personality trait, it should, theoretically, not be related to engagement with a specific DHI.

Predictive validity refers to the extent to which a score on a scale (in this case, engagement) predicts a score on a criterion measure in the future. As it is theorized that engagement influences outcomes like behavior change and clinical measures, we used the effectiveness of the DHI as this criterion measure.

### Participants

Participants were eligible for the study if they were willing to use a step counter app on their smartphones for 2 weeks. Recruitment took place via the participant pool of the University of Twente, where students receive credit for participating in research, and through the personal networks of the researchers. A total of 313 participants completed the baseline survey from December 2019 to April 2019; of the 313 participants, 288 filled out T1, 279 filled out T2, and 269 filled out T3. The 288 participants who filled out the TWEETS at least once (at T1, T2, or T3) were included in this study. Few data were missing of the included participants: T0 of all participants was complete; at T1, the number of participants that completed each measure ranged from 285 and 286 on the different measures (for example, 285 participants filled out the adherence measure while 286 filled out the TWEETS measure); at T2, the number of participants ranged from 270 and 274; and at T3, participants ranged from 259 and 265. Of the 288 included participants, most were female (228/288, 79%) and students (253/288, 88%). Most participants were German (202/288, 70%); 16% (46/288) were Dutch and 5% (14/288) were South African. In total, participants of 19 different nationalities were included. The mean age of participants was 22 (SD 7.1; range 18-70) years.

### Procedure

After participants signed up for the study and gave informed consent, they filled out the online T0 questionnaire with demographic information. After completing this questionnaire, they were asked to choose a step counter app to use for the following 2 weeks. While some apps were suggested (such as pre-installed step counter apps), participants were allowed to use any app that they preferred or already used. They were asked to open the app at least once a day, but it was suggested that it might be helpful to use the app more often, for example, during the day to check whether or not they were on track to reach their goal and then again at the end of the day to see how they did. Furthermore, participants were informed that they would receive 3 online follow-up surveys: after the first day, after a week, and after 2 weeks. It was explained that these surveys would cover their experience with using the step counter app. To gain credits for their education, participants had to complete all surveys.

### Materials

The following describes the different constructs—engagement, personality, involvement, enjoyment, usage, and perceived behavior change—that were measured in the questionnaires.

#### Engagement

Engagement was assessed with the newly developed TWente Engagement with Ehealth Technologies Scale (TWEETS; Table 1) [15]. The TWEETS consists of 9 items on a 5-point Likert scale (strongly disagree=0, disagree=1, neutral=2, agree=3, strongly agree=4). Of the 9 items, 3 are aimed at assessing behavioral engagement, 3 on cognitive engagement, and 3 on affective engagement. The full scale is presented in Table 1. The scale was adapted for each time point: after one day of usage, items were posed as expectations (for example, “*I think using this app can become part of my daily routine*”); after 1 and 2 weeks, items were posed as looking back at using the app. Furthermore, the scale allows for adaption to the studied technology by adding the technology, the goal, and the behavior relating to the goal. For the current study, this was implemented as “this app” and “increasing the number of steps I take each day.”

**Table 1:** The Twente Engagement with Ehealth Technologies Scale (TWEETS).

Item	Thinking about using [the technology] the last week, I feel that:	Construct
1	[this technology] is part of my daily routine	Behavior
2	[this technology] is easy to use^a^	Behavior
3	I'm able to use [this technology] as often as needed (to achieve my goals)	Behavior
4	[this technology] makes it easier for me to work on [my goal]	Cognition
5	[this technology] motivates me to [reach my goal]	Cognition
6	[this technology] helps me to get more insight into [my behavior relating to the goal]	Cognition
7	I enjoy using [this technology]	Affect
8	I enjoy seeing the progress I make in [this technology]	Affect
9	[This technology] fits me as a person	Affect

^a^Based on the outcomes of this study, this item was later changed to “[this technology] takes me little effort to use.”

As a second measure of engagement, the experiential subscale of the DBCI-ES (DBCI-ES-Ex) was used, consisting of 8 items on a 7-point answering scale with anchored end and middle points: not at all=1, moderately=3, extremely=7 [20]. The items were set up in the following manner: “Please answer the following questions with regard to your most recent use of the step counter app. How strongly did you experience the following?” The items were (1) Interest, (2) Intrigue, (3) Focus, (4) Inattention, (5) Distraction, (6) Enjoyment, (7) Annoyance, and (8) Pleasure, with items 4, 5, and 7 reverse-scored. In this study, Cronbach alpha values were .72, .80, and .84 on T1, T2, and T3, respectively.

#### Personality

The personality of participants was assessed with the Mini–International Personality Item Pool (Mini-IPIP), a 20-item short form of the 50-item International Personality Item Pool–5-Factor Model measure [27]. For this study, the subscales on conscientiousness and intellect/imagination were used (both consisting of 10 items with a 7-point answering scale), which have been shown to have good psychometric properties [26]. In this study, personality was assessed at T0. The Cronbach alpha value was .78 for conscientiousness and .77 for intellect/imagination.

#### Involvement

Involvement was assessed using the short version of the Personal Involvement Inventory (10 items, mean score 1-7; a higher score means more involvement), which has been shown to have good psychometric properties [23]. In this study, Cronbach alpha values were .90, .93, and .94 at T1, T2, and T3, respectively.

#### Enjoyment

Enjoyment was assessed using the enjoyment subscale of the Intrinsic Motivation Inventory (IMI, 7 items, mean score 1-7; a higher mean score means more enjoyment), which has been shown to have good psychometric properties [28]. The Cronbach alpha values in this study were .87, .91, and .92 at T1, T2, and T3, respectively.

#### Usage

As participants were allowed to use any step counter app for reasons of feasibility and ecological validity, it was not possible to gather objective usage data. Therefore, subjective usage was assessed by asking participants whether or not they opened the app at least once a day, as was advised (*yes*=adherent; *no*=nonadherent), and how often they opened the app on a regular day that they opened it (frequency). In this case, the frequency of use and adherence are largely independent of each other because the frequency relates to a regular day when they adhered (ie, opened the app at least once). Only when a user did not use the app on any given day would the user be both nonadherent and have a use frequency of 0.

#### Perceived Behavior Change

At T2 and T3, as an indication for the effectiveness of the app, we assessed whether the participants perceived behavior change due to using the step counter app. This was assessed using 1 item (“Do you feel that you have changed your behavior because of using the step counter app?”), with 3 answer options (“yes,” “maybe,” and “no”).

### Data Analyses

Analyses were performed using SSPS Statistics (version 25; IBM Corp). Analyses were conducted with the data of the participants who completed the measures that were used in each specific analysis. Due to the limited amount of missing data, no imputation of missing values was required. Engagement measured by the TWEETS and by the DBCI-ES-Ex violated the assumption of normality at all time points by being left-skewed. However, due to the relatively large sample size and the robustness of analyses such as Pearson correlations and analyses of variance (ANOVAs), parametric tests were used [29-31]. Furthermore, we performed exploratory nonparametric analyses on our data to see whether the results from these analyses would lead to different conclusions. This was not the case, and therefore, we report only the parametric analyses in this paper.

Internal consistency of the TWEETS and the DBCI-ES-Ex was assessed using the Cronbach alpha value of each scale at T1, T2, and T3. A value of .7 was seen as the absolute minimum, while .8-.9 was seen as good internal consistency [32,33]. Exploratory factor analysis for the TWEETS was performed using SPSS' Maximum Likelihood (ML) method with oblique rotation (SPSS' direct oblimin) for all time points separately [34]. The Scree test (by examining the scree plots) combined with an assessment of eigenvalues was used to determine the number of factors [34]. Test-retest reliability for the TWEETS and DBCI-ES-Ex were assessed by examining Pearson correlations between the TWEETS scores on T1, T2, and T3, and by examining Pearson correlations of the DCBI-E scores on T1, T2, and T3. Although no standards exist for a minimum acceptable value for a test-retest reliability estimate [35], a correlation coefficient does provide information on the stability or variation of a scale over time. Convergent validity was assessed by calculating Pearson correlations between the TWEETS, DBCI-ES-Ex, involvement, enjoyment, and use frequency at T1, T2, and T3. Moreover, differences in the TWEETS and DBCI-ES-Ex between participants that adhered and did not adhere to the app were calculated using a one-way ANOVA. Divergent validity was assessed by calculating Pearson correlations between the TWEETS and personality (conscientiousness and intellect/imagination) and between the DBCI-ES-Ex and personality (conscientiousness and intellect/imagination). Lastly, predictive validity was assessed by separate linear regression analyses using TWEETS and separate linear regression analyses using DBCI-ES-Ex on T1 to predict perceived behavior change at T2 and T3, and TWEETS and DBCI-ES-Ex at T2 to predict perceived behavior change at T3.

## Results

### Internal Consistency and Reliability

Table 2 shows the mean values of the scores on the TWEETS and the DBCI-ES-Ex at the various time points. The Cronbach alpha values of the TWEETS were .86, .86, and 0.87 on T1, T2, and T3, respectively, indicating good internal consistency. Exploratory factor analyses of the TWEETS indicated that a 1-factor structure best fits the data and explained 41.4% of the variance, based on observed data on T1. Data from T2 and T3 also indicated a 1-factor structure with similarly explained variance at T2 (42.2%) and somewhat less explained variance at T3 (30.1%). Of the 9 items, 8 items loaded strongly (> 0.5) on the 1 factor. The other item (“this technology is easy to use”) loaded 0.35 - 0.39 on the factor on the different time points. This is still seen as acceptable to retain an item in a scale, as it is above the minimum threshold of 0.32 [30]. Furthermore, the Cronbach alpha values with this item removed showed minimal differences in internal consistency (.86, .87, and .88 on T1, T2, and T3, respectively). Pearson correlations of the TWEETS at different time points were all significant (*P*<.001), with values of 0.58 (T1-T2), 0.61 (T1-T3), and 0.74 (T2-T3), showing moderate test-retest reliability. Pearson correlations of the DBCI-ES-Ex at different time points were all significant (*P*<.001), with values of 0.70 (T1-T2), 0.67 (T1-T3), and 0.78 (T2-T3), showing relative stability over time.

**Table 2:** Descriptive statistics for the TWente Engagement with Ehealth Technologies Scale (TWEETS) and the experiential subscale of the Digital Behavior Change Intervention Engagement Scale (DBCI-ES-Ex) at different time points.

Scale	Time point 1, n; mean (SD)	Time point 2, n; mean (SD)	Time point 3, n; mean (SD)
TWEETS	286; 2.85 (0.63)	274; 2.65 (0.65)	259; 2.65 (0.65)
DBCI-ES-Ex	286; 4.64 (0.74)	273; 4.50 (0.86)	266; 4.49 (0.90)

### Convergent Validity

Pearson correlations between the TWEETS, DBCI-ES-Ex, involvement, enjoyment, and use frequency at the different time points are shown in Table 3. The 2 engagement scales showed moderate to strong correlations with each other. Both the TWEETS and DBCI-ES-Ex showed significant moderate to strong correlations with involvement and enjoyment, which become stronger at later time points. The DBCI-ES-Ex scale consistently showed stronger correlations than the TWEETS with both involvement and enjoyment, with particularly strong correlations with enjoyment. Both scales show no or weak correlations with reported use frequency.

**Table 3:** Pearson correlations between the TWente Engagement with Ehealth Technologies Scale (TWEETS), the experiential subscale of the Digital Behavior Change Intervention Engagement Scale (DBCI-ES-Ex), involvement, enjoyment, and use frequency.

Scale & time point			Involvement	Enjoyment		Use frequency		DBCI-ES-Ex
**TWEETS**								
	T1	0.57^a^		0.60^a^	0.08		0.57^a^	
	T2	0.61^a^		0.70^a^	0.24^a^		0.73^a^	
	T3	0.67^a^		0.77^a^	0.04		0.80^a^	
**DBCI-ES-Ex**								
	T1	0.54^a^		0.69^a^	0.02		N/A^b^	
	T2	0.63^a^		0.81^a^	0.26^a^		N/A^b^	
	T3	0.70^a^		0.82^a^	0.03		N/A^b^	

^a^Significant with *P*<.001.

^b^N/A: not applicable.

**Figure 1:**
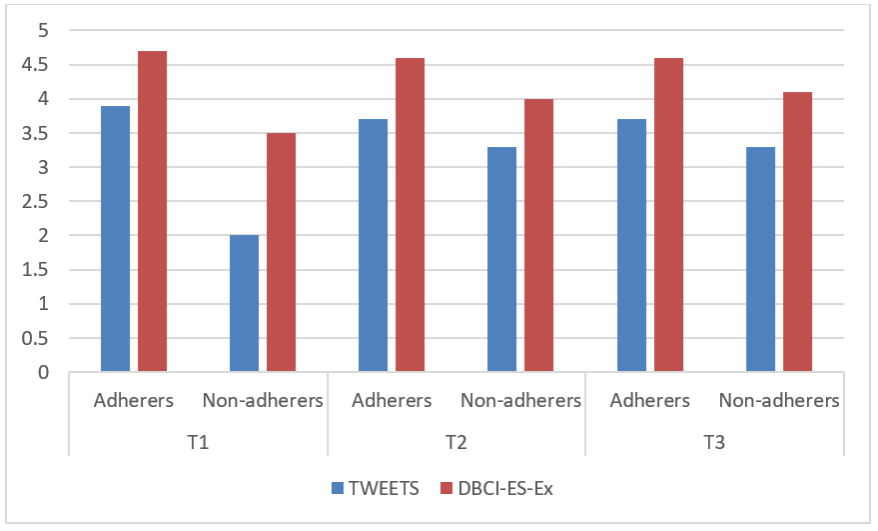
Engagement scores of adherers and nonadherers at the different time points. DBCI-ES-Ex: the experiential subscale of the Digital Behavior Change Intervention Engagement Scale; T: time point; TWEETS: TWente Engagement with Ehealth Technologies Scale.

Lastly, mean scores on the TWEETS between participants that adhered to the app (using it at least once a day) and those that did not adhere significantly differed at all time points (*P*<.001), with adherers scoring higher than nonadherers (Figure 1). The same was true for the DBCI-ES-Ex scale, with *P*=.028 at T1 and *P*<.001 at T2 and T3. However, the number of nonadherers was low, especially at T1 (T1: 2/285; T2: 36/270; T3: 46/262).

### Divergent Validity

Pearson correlations between conscientiousness and the TWEETS at the different time points were nonsignificant. The same was true for intellect/imagination. For the DBCI-ES-Ex, the Pearson correlation with conscientiousness at T1 was weak but significant (0.17, *P*<.01). Pearson correlations at the other time points and with intellect/imagination were nonsignificant.

### Predictive Validity

Results of the linear regression analyses using the TWEETS and DBCI-ES-Ex to predict perceived behavior change are shown in Table 4. These results show that both the TWEETS and DBCI-ES-Ex demonstrate predictive validity. As can be expected, the shorter the time between the measurement of engagement and the measurement of perceived behavior change, the more predictive engagement is, with engagement at T2 explaining around 24% of the variance of perceived behavior change at T3, and engagement at T1 explaining 12-14% of the variance of perceived behavior change at T2. However, engagement measured at T1 was also predictive of perceived behavior change at T3, with an explained variance of 16% when using the TWEETS and 9% using the DBCI-ES-Ex.

**Table 4:** Regression analyses with the TWente Engagement with Ehealth Technologies Scale (TWEETS) and the experiential subscale of the Digital Behavior Change Intervention Engagement Scale (DBCI-ES-Ex) to predict perceived behavior change.

Scale & time point	Perceived behavior change T2			Perceived behavior change T3			
	Beta	R^2^	*F* ^a^		Beta	R^2^	*F* ^a^
TWEETS T1	-.38	0.144	45.34		-.40	0.163	51.36
TWEETS T2	N/A^b^	N/A^b^	N/A^b^		-.49	0.242	84.04
DBCI-ES-Ex T1	-.35	0.120	35.84		-.30	0.087	25.70
DBCI-ES-Ex T2	N/A^b^	N/A^b^	N/A^b^		-.50	0.244	84.69

^a^*P*<.001

^b^N/A: not applicable.

## Discussion

### Principal Findings

This study set out to evaluate the psychometric properties of the new TWEETS. A secondary exploratory objective was to compare these psychometric properties to those of the experiential subscale of the DBCI-ES.

For the TWEETS, internal consistency was high, indicating that the different items of the scale all measure the same construct. In line with this, exploratory factor analyses showed the scale consists of 1 factor; we did not find the 3-factor structure that we expected, resulting from the behavioral, cognitive, and affective engagement components and their respective items in the scale. An explanation could be that engagement as a concept comprises only 1 component and not the proposed 3 components. However, because of the theoretical background of the concept, we would be hesitant to accept this explanation without further research. A more likely explanation might be that there is a theoretical overlap between the component; for example, enjoying seeing the progress that you make through an app (affective engagement) might also influence the extent to which you feel that the app motivates you to reach your goals (cognitive engagement). Also, behavioral engagement might be more of a consequence of cognitive and affective engagement (for example, because people feel that the technology helps them achieve their goals and is fun, they might establish a routine of using it), rather than being on the same level as these components. However, an explanation might also lie in the small number of items in the scale; 5 or more strong loading items per factor are seen as desirable [34], which indicates that we should have at least 15 items that would be retained in the scale, indicating an even higher number of a priori items. However, data showed that the 1-factor structure that was observed in the data was solid, with 8 items loading strongly and the last one above the minimum to retain an item in a factor [34]. Future research could explore whether the components can be measured as separate constructs, for example, by adding more items and examining whether this is desirable, as it would also increase the length of the scale and, therefore, its participant burden. Another option may be to retain the TWEETS as a short-form engagement measure and develop separate measures for the components to be able to investigate more detailed patterns of engagement when in-depth analyses are needed, for example, to explore whether different people have, or different technologies induce, a different distribution of engagement over the components. What the 1-factor structure of the TWEETS does show is that the different components of engagement are indeed related and measure the same construct. This strengthens the assumption that engagement is a combination of behavior, cognition, and affect, and more than just usage behavior.

Test-retest reliability analyses showed moderate positive correlations between the first and later time points, and a strong positive correlation between the later time points. Although this may be interpreted as low test-retest reliability when looking at this first time point, this might not be surprising, as engagement after 1 day of usage might be initial engagement, which may be different from longer-term engagement. This is also reflected in different models on the process of engagement in which engagement may vary over time [8,17]. It seems reasonable to expect engagement to change after getting more acquainted with the app, and when actually trying to use the app for a longer period in daily life. As integrating technology in daily life is often one of the largest struggles in eHealth and specifically DHIs [36], being able to do so or not might be an obvious reason for a change in engagement. Taking these factors into account, it may be even more remarkable to see that participants’ initial engagement, based on only 1 day of using a DHI, is still as related to engagement at a later stage. Additionally, this change in the level of engagement over time might also explain the somewhat differing findings on convergent and divergent validity of the engagement scales at different time points. As is seen in other studies, different aspects might be more important and more related to engagement in the different stages of the process of engagement [17].

Related to this, the TWEETS showed predictive validity: engagement at an earlier time point was able to predict perceived behavior change at a later time. Even the engagement measure after the first day of using a technology showed this capability, which opens up many interesting possibilities [13]; when we are able to assess early on in an intervention whether or not we expect it is going to be effective for an individual, we may be able to direct more support to those for whom it might not be effective. This can be, for example, in the form of personalized feedback or the suggestion of using a different intervention.

Analyses of divergent validity showed that the TWEETS was not correlated with personality factors. This strengthens the assumption that engagement is a separate construct and that this is reflected in this new scale. Analyses of convergent validity showed that the TWEETS is moderately to strongly correlated with involvement (which is seen as theoretically related to cognitive engagement) and enjoyment (which is seen as theoretically related to affective engagement) [24]. The strength of the correlations indicate that the concepts are related but should be viewed as separate. For behavioral engagement, we choose to relate the TWEETS to adherence and frequency of use, as these are aspects that are often seen as related to engagement [9,14,24], or even used to assess criterion validity [19]. Correlations between the engagement measure and frequency of use were nonsignificant or small, while differences between adherers and nonadherers on engagement were significant but small. This might indicate that engagement is related to usage, but much less so than is often assumed, again strengthening the multiple component definition of engagement. However, it should be noted that our usage measure was subjective, which sheds some doubt on the robustness of this measure. This could provide an alternative explanation of the weak correlation between frequency of use and engagement. Future research could investigate whether objectively measured frequency of use should be seen as a measure of convergent validity or whether it could be more appropriate to see it as a measure to assess divergent validity.

The exploratory comparison of the psychometric properties of both engagement scales—the TWEETS and the DBCI-ES-Ex—revealed that both scales show similar properties. As could be expected, the scales are significantly correlated to each other. Interestingly, this correlation is moderate at the first time point but strong at the last time point. This might indicate that they vary on how much they take the first impression that a DHI makes into account. A few notable differences between both scales are that the DBCI-ES-Ex shows quite a strong correlation to enjoyment, a bit stronger than the TWEETS, and that DBCI-ES-Ex did not fully reflect divergent validity. This might indicate that the DBCI-ES-Ex captures engagement not as a unique concept but more like enjoyment. Additionally, the TWEETS shows more predictive validity between the first and the last time point compared to the DBCI-ES-Ex. Overall, the TWEETS seems to reflect engagement better as a multifaceted and unique concept that has predictive value for the outcomes of an intervention.

### Limitations

This study has a few limitations that need to be discussed. First, the study was done mostly with students who received credits for participating in the study and thus for using the step counter app, which might have influenced their usage and engagement. However, as we were interested in the relationships between engagement and other measures, and not the level of engagement or usage, we feel that studying this target group was an appropriate first step. Furthermore, we needed data on a broad spectrum of engagement, including low engagement. Therefore, including a target group that might not be completely intrinsically motivated to use a DHI was seen as appropriate. Future research should focus on measuring engagement in different populations and settings, for example, with lower educated participants or within the context of regular (mental) health care. It would be interesting to see whether different populations show different styles of engagement, such as whether lower educated participants might be more often affectively engaged, and whether this impacts the factor analysis of the scale.

Second, we used self-reported usage data and our variable for predictive validation was perceived behavior change and not actual behavior change. It would have been preferable to use objective usage data and actual behavior change data, but this was deemed infeasible for this exploratory study because participants could use any step counter app for reasons of ecological validity and feasibility. Therefore, it was not possible to objectively collect the usage data of all these different apps or the number of steps participants took every day. Also, to be able to measure actual behavior change in the average number of steps per day, a larger data collection period would have been necessary, which was beyond the scope of this study. However, as this was an exploratory study that provides a first step towards establishing whether the TWEETS is a useful measure of engagement in DHIs, this approach was deemed appropriate. Based on the promising findings of this study that engagement was able to predict perceived behavior change, the next step is to validate this finding in future studies using engagement to predict actual behavior change and other intervention outcomes. Also, the direction of change was not explicitly stated in the question. However, at multiple points in the study, it was stated that the goal of the app was to increase the number of steps taken per day. Moreover, participants could indicate in what way they changed their behavior. All participants that filled out that question indicated that they took more steps per day and not less steps. Therefore, we feel that the impact of not explicitly stating the direction of change was negligible.

A final limitation is that 1 item of the scale performed a bit less well than the others, although still above the minimum threshold to retain an item in a scale: the item “*this technology is easy to use*” should be revised in a way that better reflects the behavioral component of engagement and is less related to usability, which can be seen as a predictor of engagement. A different way to state the item that better reflects the quality of engagement behavior from our definition could be “*this technology takes me little effort to use*.” Nonetheless, the scale performed quite well on most criteria, indicating the potential of this new instrument.

### Conclusion

Overall, the TWEETS seemed to perform quite well as a 1-factor engagement measure: the scale showed high internal consistency, reasonable test-retest reliability and convergent validity, good divergent validity, and reasonable predictive validity. These properties seem comparable to—and, on some aspects, somewhat superior to—those of the experiential subscale of the DBCI-ES. Further research is needed to replicate these findings in other target groups and eHealth technologies; however, the TWEETS seems to be a valuable addition to the toolbox of eHealth researchers, allowing them, amongst others, to be able to better investigate the relationship between engagement and effectiveness.

Lastly, the psychometric quality of a scale is, of course, a reflection of how closely a scale matches the conceptualization of the concept. Therefore, this paper can also be seen as an attempt to conceptualize and define engagement as a unique concept, different from, for example, adherence and user experience. We have argued for a definition of engagement that entails behavioral, cognitive, and affective components and, through the analyses in the paper, have provided arguments in favor of this definition. This paper provides a first step toward an acceptable standard of defining and measuring engagement.

## Abbreviations

ANOVA: analysis of variance
DBCI-ES: Digital Behavior Change Intervention Engagement Scale
DBCI-ES-Ex: experiential subscale of the Digital Behavior Change Intervention Engagement Scale
DHI: digital health interventions
TWEETS: TWente Engagement with Ehealth Technologies Scale
